# The Role of Oxidative Stress in Hypertension: The Insight into Antihypertensive Properties of Vitamins A, C and E

**DOI:** 10.3390/antiox13070848

**Published:** 2024-07-15

**Authors:** Ewelina Młynarska, Laura Biskup, Maria Możdżan, Olivia Grygorcewicz, Zofia Możdżan, Jan Semeradt, Michał Uramowski, Jacek Rysz, Beata Franczyk

**Affiliations:** 1 Department of Nephrocardiology, Medical University of Lodz, Ul. Zeromskiego 113, 90-549 Lodz, Poland; 2 Department of Nephrology, Hypertension and Family Medicine, Medical University of Lodz, Ul. Zeromskiego 113, 90-549 Lodz, Poland

**Keywords:** hypertension, blood pressure reduction, oxidative stress, dietary antioxidants, vitamin C, vitamin E antioxidative properties of vitamins, vitamin A, beta-carotene, carotenoids

## Abstract

Hypertension stands as a pervasive global health challenge, contributing significantly to mortality rates worldwide. Various factors, including lifestyle choices and dietary habits, contribute to the development of hypertension. In recent years, oxidative stress has garnered significant attention as a factor influencing hypertension risk, prompting a shift in research focus towards exploring it as a potential target for prevention and treatment. Antioxidants found in our diet, such as vitamins C, E and carotenoids exhibit the ability to neutralize reactive oxygen species, thereby mitigating oxidative stress. In addition, Vitamin A has an antioxidant effect despite not being an antioxidant itself. Consequently, supplementation or increased intake of these antioxidants has been hypothesized to potentially lower blood pressure levels and aid in the management of hypertension, thereby potentially prolonging life expectancy. Research findings regarding this effect have been diverse. This paper examines the existing literature demonstrating favorable outcomes associated with antioxidant supplementation.

## 1. Introduction

Hypertension (HT), characterized by continuous elevation in blood pressure (BP), is a widespread chronic disorder impacting people across diverse demographics and geographic regions. With approximately 1.28 billion people affected globally, it firmly solidifies itself as one of the most common non-communicable diseases with significant health implications [1]. Often referred to as a ‘silent killer’ due to its typically asymptomatic nature, HT is a primary risk factor for premature cardiovascular morbidity and mortality worldwide. Despite its global presence, awareness and control of HT remain inadequate, with an estimated 46% of affected individuals unaware of their condition. This lack of awareness contributes to the alarming statistic that only about 21% of hypertensive individuals have their BP adequately controlled [2]. Uncontrolled HT is a leading risk factor for cardiovascular diseases (CVD), including coronary heart disease and stroke, which together account for nearly 18 million deaths annually [1]. It is worth noting that a reduction in systolic blood pressure (SBP) by even 1 mmHg could potentially prevent up to 10,000 deaths related to coronary heart disease annually [3].

HT develops due to a multitude of factors. Central contributors include the overactivity of the renin-angiotensin-aldosterone system, which increases blood volume and vascular resistance, and the heightened activation of the sympathetic nervous system, leading to an elevated heart rate and vasoconstriction. Endothelial dysfunction also plays a significant role by impairing vasodilation. Recently, oxidative stress (OS) has gained more attention as a critical factor in the development of HT [4]. OS is a key factor in the onset of numerous diseases, such as atherosclerosis, vascular disorders, obesity and metabolic syndrome [5]. It is defined as the predominance of oxidants over antioxidants, which leads to a disturbance in redox signaling and molecular damage. Reactive oxygen species (ROS), produced mainly in mitochondria, regulate various cellular functions such as inflammation and stress response. Elevated ROS levels induce endothelial damage, cardiovascular remodeling, renal dysfunction, sympathetic nervous system excitation, immune cell activation and systemic inflammation, which makes them important in the pathophysiology of HT. Moreover, OS disrupts nitric oxide (NO) bioavailability, a key regulator of vascular tone, leading to impaired endothelium-dependent vasodilation and further exacerbating HT [6].

## 2. Methods

In this review, we searched the National Library of Medicine’s PubMed for studies published after 1996 written in English. The searched keywords were as follows: “vitamin A”, “beta carotene”, “carotenoids”, “vitamin C”, “vitamin E”, “hypertension”, “primary hypertension”, “vitamin supplementation”, “vitamin intake”.

The inclusion criteria were as follows: (1) participants over 18 years old, both sexes, (2) studies that investigated the relation between serum vitamins A, C and E and blood pressure among hypertensive subjects or normotensives, (3) studies that investigated the relation between vitamins A, C and E supplementation and blood pressure among hypertensive subjects or normotensives, (4) meta-analyses, randomized control trials and observational articles including cross-sectional studies, case-control studies and cohort studies.

We also included studies performed on specific groups of participants: pregnant women and diabetic patients.

The exclusion criteria were as follows: (1) duplicate publications, (2) case reports, reviews, non-clinical tests and animal experiments, (3) studies included less than 50 participants, (4) studies reported no effects on SBP and DBP changes.

**Figure 1:**
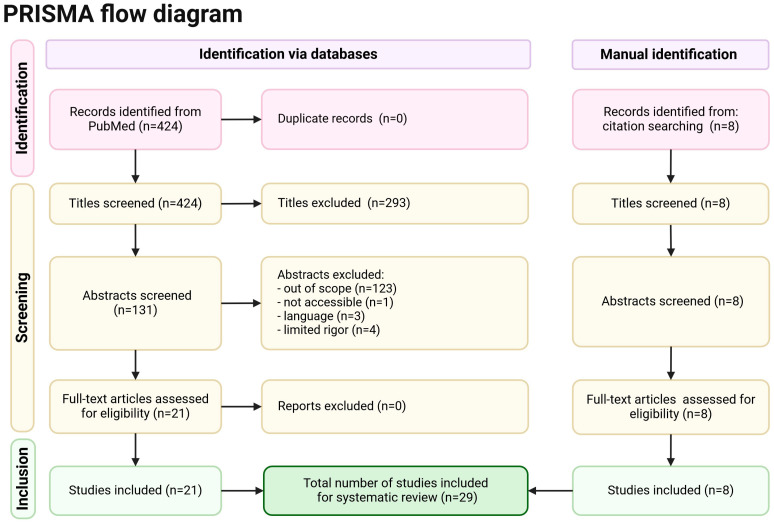
PRISMA flow diagram with exclusion criteria for the selection of sources for the purpose of the review [7].

The PRISMA flow diagram is shown in Figure 1.

## 3. Role of Oxidative Stress in Hypertension

Oxidative stress is characterized by the exceeding generation of ROS compared to the antioxidant system, which is caused either by increased production of ROS or a decrease in the antioxidants [8].

ROS are produced by cells as the result of the metabolism of molecular oxygen (O_2_) [8]. In physiologic conditions, ROS are important for redox regulation in maintaining the integrity of endothelium and vascular function [9]. In pathologic situations, excessive ROS generation can cause cell injury or death by reacting with cellular macromolecules, such as RNA, DNA, proteins, lipids and carbohydrates [10].

Multiple external factors can affect ROS production including high-fat diet, cigarette smoke, consumption of alcohol, air pollution and cancer radio- and chemotherapy [11,12].

ROS are a significant factor in the development of CVDs and the regulation of vascular and cardiac function. The most important ROS in the cardiovascular system are O_2_, H_2_O_2_, NO and ONOO- [9].

O_2_- is generated through partial reduction of O_2_ and is a key component in generating other reactive species [13]. In vessels, O_2_- increases the contraction and proliferation of smooth muscle cells. It also increases the chemoattraction of inflammatory cells [14]. O_2_- is reduced by superoxide dismutase to H_2_O_2_ [13]. Numerous mechanisms have described linking H_2_O_2_ to HT, including vascular contraction, vascular hypertrophy and hyperplasia, decreased diuresis and natriuresis and increased spinal sympathetic outflow. H_2_O_2_ vascular and urinary levels are also elevated in renin-angiotensin-mediated HT [15]. Nonetheless, in some circumstances, H_2_O_2_ can also have vasodilatory properties via oxidative activation of protein kinase G1-α and smooth muscle relaxation [16].

NO is synthesized by endothelial nitric oxide synthase (eNOS) and is a vasodilator that increases blood flow by causing the relaxation of smooth muscles. Moreover, NO can prevent the adhesion of platelets (PLTs) and leukocytes to the walls of blood vessels [17]. NO and O_2_- can interact, resulting in the decrease of NO bioavailability, which promotes vasoconstriction [14]. ONOO- is the product of the reaction between O_2_- and NO and is a strong, highly unstable oxidant [18]. ONOO- can oxidize the tetrahydrobiopterin (BH4), the cofactor of eNOS. In this process, the eNOS enzyme becomes uncoupled and starts producing O_2_- instead of NO [19]. This process leads to endothelial dysfunction and elevated BP partially due to vasoconstriction [20].

A major source of ROS generation in the cardiovascular system is a family of nicotinamide adenine dinucleotide phosphate oxidase (Nox) [6]. There are seven isoforms of the Nox with Nox1, Nox2, Nox4 and Nox5 that might be involved in the development of HT [21]. Mice with Nox 1 overexpression, when subjected to chronic angiotensin II (Ang II) infusion (a potent vasoconstrictive peptide), exhibited significantly elevated BP. Conversely, mice deficient in Nox1 showed moderately reduced baseline BP and demonstrated resistance to Ang II-induced hypertension [22,23,24]. Mice without the Nox2 enzyme also showed lesser BP elevation and O_2_- production in response to Ang II infusion [24]. The studies on Nox4’s role in HT yielded mixed results. In one study, endothelium-specific overexpression of Nox4 resulted in decreased BP through H_2_O_2_-mediated vasorelaxation [24,25]. However, other studies conducted on mice with deletion of Nox4 showed either declined or no response in BP after Ang II infusion, compared to wild mice. These results may suggest Nox4’s involvement in the initiation of HT, induced by Ang II [24]. In human diabetic nephropathy overexpression of Nox5 in the podocytes can modify SBP and alter filtration barrier function [26].

ROS production in the kidney can also be attributed to the development of HT. An increase in O_2_- in the afferent arteriole or in the macula densa inactivates NO, leading to afferent arteriolar vasoconstriction and a glomerular filtration rate reduction. In addition, activation of the Nox in the renal medulla can reduce natriuresis and increase BP [19]. OS can also trigger podocyte and mesangial cell autophagy and death, profibrotic pathways and glomerulosclerosis promotion as well as disruption of the glomerular filtration barrier, which results in proteinuria. ROS can also increase mesangial matrix formation and renal fibrosis in mammals by upregulation of transforming growth factor-β [27].

The immune system also has its role in the pathogenesis of HT. Mice lacking lymphocytes had a lower hypertensive response to Ang II. Patients with HT also typically have higher levels of circulating immunosenescent proinflammatory CD8+ T cells and CD4+ cells. CD8+ T cells produce tumor necrosis factor α (TNF-α) [10], which is linked to the pathogenesis of HT through a not clearly defined neural mechanism, presumably through excitation of the sympathetic nervous system [28]. Moreover, TNF-α inhibitors can reduce clinical symptoms of HT [29]. CD4+ cells produce IL-17a, which promotes renal and vascular impairment that can lead to BP elevation. Superoxide anion production by Nox in dendritic cells (DCs) leads to the creation of highly reactive isolevuglandins (IsoLGs). IsoLGs bind to the body’s proteins, which are then presented as new antigens by DCs. This triggers an immune response, ultimately causing dysfunction in the kidneys and blood vessels leading to HT. In addition, DCs that accumulate IsoLGs, produce cytokines that promote IL-17a-producing cell differentiation [30].

**Figure 2:**
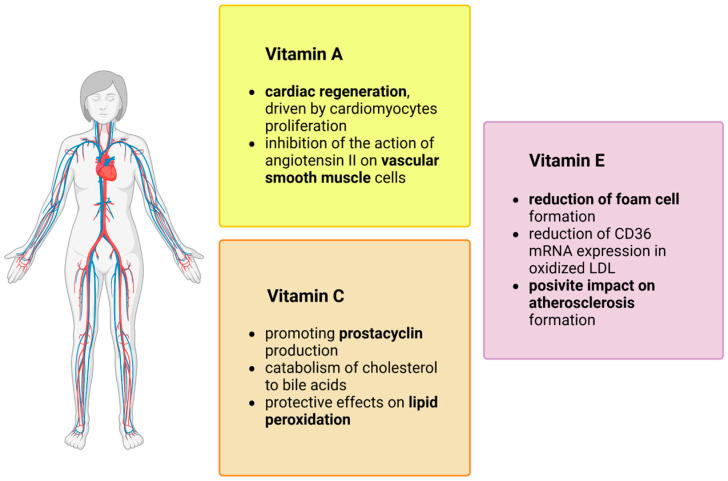
The molecular mechanisms of vitamins with antioxidative properties in the cardiovascular system [33,34,35,36].

Excessive ROS generation and subsequent oxidative damage can be detrimental to the health of the cells. In order to counter this process, cells have evolved a protective mechanism that prevents ROS accumulation and maintains redox status. This cellular antioxidant system consists of enzymes including superoxide dismutase, glutathione peroxidase, catalase, thioredoxin-peroxiredoxin [10] and nonenzymatic antioxidants like vitamins C and E [31,32]. These vitamins as well as Vitamin A exhibit numerous beneficial effects on cardiovascular health through various mechanisms, as shown in Figure 2.

## 4. Antihypertensive Properties of Vitamins

### 4.1. Vitamin A and Provitamin A

The elevated serum level of vitamin A’s provitamin, *β*-carotene, seems to be associated with decreased BP [37]. The study of Fazal et al. revealed that *β*-carotene particularly has an impact on the expression level of the *angiotensin-converting enzyme (ACE)* gene, whose function plays a role in the pathogenesis of HT [38]. In addition, carotenoids exert an effect on protecting endothelial cells from oxidation and cellular damage, because of their activity towards ROS [39,40]. Furthermore, carotenoids inhibit tissue factor activity and gene expression in human endothelial cells, which is associated with a lower risk of thrombosis [41].

### 4.2. Vitamin C

Vitamin C exhibits hypotensive properties through various mechanisms. Firstly, it enhances the production of vasodilators, such as NO via increasing intracellular levels of tetrahydrobiopterin, a cofactor for endothelial NO synthase [42].

Moreover, vitamin C facilitates diuretic sodium excretion and cytosolic calcium level reduction by blocking the Ca channel, which also contributes to BP reduction [43]. Various factors influence vitamin C, particularly glutathione (GSH), which takes part in ascorbic acid recycling and increases its antioxidant effect [44]. Additionally, by inhibiting *ACE-2* gene expression, vitamin C lowers BP levels, making this gene a potential target for antihypertensive therapy [45].

### 4.3. Vitamin E

Vitamin E also presents various antihypertensive properties. It enhances the synthesis of prostaglandin I2 by simultaneously inhibiting the activities of phospholipase A2 and cyclooxygenase-2 [46]. Therefore, it helps to decrease arterial stiffness and uphold the function of the vascular endothelium [47]. Furthermore, vitamin E has the ability to inhibit smooth muscle cell proliferation, consequently preventing aortic damage.

In addition, vitamin E effectively reduces the inflammatory response. This action is manifested by the reduction of serum interleukin-6 (IL-6) levels, thereby exhibiting potent anti-inflammatory effects [48].

Vitamin E has a significant antioxidant effect as it neutralizes ROS, which also has an impact on reducing the risk of developing HT [49]. Vitamin E supplementation has also been observed to reduce plasma levels of F2-isoprostanes, which are formed by free radical attack on arachidonic acid. One of the F2-isoprostanes, 15-F2t-isoprostane, has a regulatory effect on BP. Its role is described as a renal vasoconstrictor, reducer of glomerular filtration and renal blood flow. Moreover, it can affect vascular tone by releasing the vasoconstrictor endothelin-1 from aortic endothelial cells. These actions lead to the elevation of blood pressure [50].

## 5. Vitamins’ Characteristics and Their Dietary Sources

**Figure 3:**
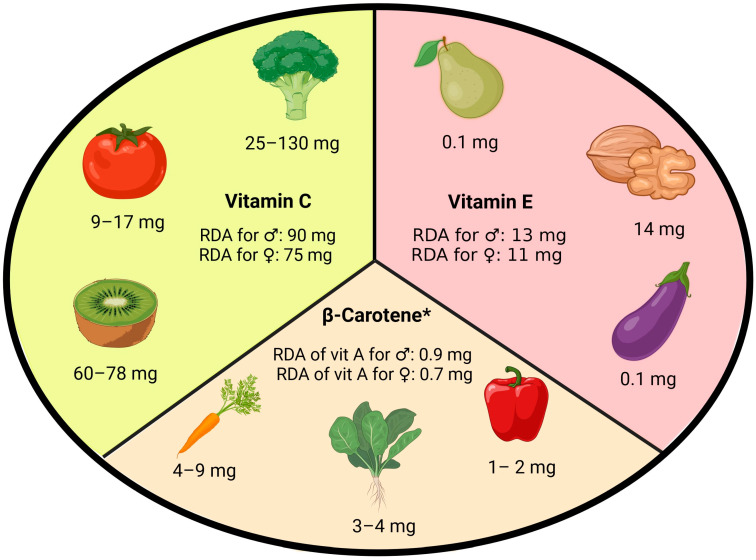
Dietary vitamin content (mg/100 g fresh weight) and its recommended daily allowance (RDA). Based on studies of Doseděl et al. [52], Carazo et al. [53] and Zhang et al. [54]. * Vitamin A intake recommendation is usually expressed as retinol activity equivalent (REA), where 1 REA equals 1 µg of retinol equals, 12 µg of β-carotene and 24 µg of α-carotene or β-cryptoxanthin.

Vitamins, especially those with antioxidant properties, have wide-ranging effects on many CVDs. Studies have demonstrated a correlation between higher intake of vitamin-rich dietary sources, particularly those abundant in vitamins A, C and E, and lower cardiovascular risk [51]. Products rich in these vitamins and their recommended daily allowance (RDA) are shown in Figure 3.

### 5.1. Vitamin A

Vitamin A consists of fat-soluble retinoids, which include retinol, retinal and retinyl esters [55]. There are also several carotenoids, the most prevalent of which is *β*-carotene [56]. Upon ingestion, both retinol and *β*-carotene undergo oxidation processes within tissues, converting into retinal and retinoic acid [57,58]. The metabolites of vitamin A play crucial roles in biological functions, which include epithelial barrier integrity, the reproductive system and immune responses [58].

The functions of vitamin A are multifaceted. Vitamin A is not approved as a direct antioxidant, although it presents with antioxidant activity. It has a well-documented impact on gene expression, particularly in genes involved in the cellular response to oxidative stress. Vitamin A is involved with their transcription factors as a ligand. It activates the Keap1/Nrf2/ARE pathway, causing the induction of phase 2 enzymes, which as opposed to direct antioxidants, are long-lived and as catalysts are not consumed [59]. In addition, vitamin A acts as a precursor for retinoid X receptors and for ligands for retinoic acid receptors [60]. These receptors act as transcription factors that modulate the expression of target genes, including those encoding antioxidant enzymes [61,62].

In addition, carotenoids have the ability to scavenge singlet oxygen and peroxyl radicals [63]. Beta-carotene plays a role in delaying the radical propagation chain generated by lipid oxidation. Due to this ability, beta-carotene is classified as a membrane antioxidant. The antioxidant activity of carotenoids in vivo is observed in LDLs and cellular membranes [64]. Their actions take place through nuclear factor erythroid 2-related factor 2 and peroxisome proliferator-activated receptor, which downregulate oxidative stress [65,66]. Animals are incapable of de novo vitamin A synthesis. Preformed vitamin A, such as retinyl palmitate, is predominantly sourced from animal products, such as eggs and milk. Provitamins are obtained from plant-based sources, including carrots, mangoes, oranges, tomatoes and spinach [67,68]. Cooking vegetables breaks down their cellular structures, enhancing nutrient availability. Pairing cooked vegetables with dietary fat, such as olive oil, is crucial for the absorption of vitamin A in the intestine [69].

It is recommended that adult males consume 900 µg of vitamin A per day, while adult females should aim for 700 µg [70]. In clinical aspects, low plasma retinol levels serve as an indicator of its significant deficiency. A plasma retinol concentration of 20 µg/dL or lower is indicative of moderate vitamin A deficiency (VAD), while a level of 10 µg/dL or less is considered severe [71].

Consequences of VAD include xerophthalmia, heightened susceptibility to infections, stunted growth and anemia [72]. Individuals deficient in vitamin A are at increased risk of complications from diseases such as diarrhea and respiratory infections [72]. VAD is particularly concerning in the context of measles, a significant cause of childhood morbidity and mortality [73]. It is also associated with an increased risk of tuberculosis [74] and can impair immune function due to elevated levels of inflammatory cytokine [75]. Vitamin A supplementation is beneficial in treating conditions such as measles, xerophthalmia and severe malnutrition. Additionally, it also plays a crucial role in preventing deficiencies in pregnant women and children residing in areas endemic to VAD [76].

A protective effect of vitamin A on cardiovascular outcomes was noted. Its supplementation was associated with a lower risk of coronary heart disease and ischemic heart disease. Higher plasma *β*-carotene significantly correlated with a lower incidence of cardiac events, while low levels were associated with a higher risk of stroke [63]. Moreover, low plasma vitamin A level was found in patients after myocardial infarction [77].

### 5.2. Vitamin C

Vitamin C, known as ascorbic acid, is a vital water-soluble simple carbohydrate, which can be supplied to the body only through food and supplementation, as humans have lost the ability to produce it [78]. It is well-known for its antioxidative properties and serving as a cofactor in various enzymatic reactions. As an antioxidant, ascorbic acid takes part in modulating the levels of ROS and enhancing the efficacy of other antioxidants [79]. It takes part in ROS regulation by inhibiting Nox and inducible NOS, exerting influence in the neutralization of free radicals within the plasma, cytosol and extracellular fluid compartments [79]. Vitamin C is a substrate of the ascorbate peroxidase and prevents the adhesion of phagocytes to endothelium and concomitant endothelial damage through ROS [79]. Furthermore, it helps to regenerate oxidized vitamin E, thereby replenishing its antioxidative capacity [80]. These functions are facilitated by its electron-donating ability to donate electrons [81].

Vitamin C is a crucial component of various physiological processes, encompassing the synthesis of catecholamines (such as dopamine, epinephrine or norepinephrine) as well as the production of cortisol and vasopressin [82]. It also impacts the functioning of B- and T-lymphocytes and collagen biosynthesis [79]. Moreover, it significantly contributes to the regulation of iron metabolism by binding and reducing Fe^3+^ to Fe^2+^ ions, influencing its uptake from dietary sources [83].

Vitamin C is found in two forms: ascorbic acid and dehydroascorbic acid [84]. Both possess antiscorbutic properties, as cells convert dehydroascorbic acid into the biologically active reduced form, ascorbic acid [85]. Overall, the human body contains approximately 20 milligrams of vitamin C per kilogram of body weight [52]. Plasma adequate levels oscillate nearly 50 μmol/L with hypovitaminosis defined as less than 23 μmol/L and deficiency as less than 11 μmol/L [86]. Symptoms of vitamin C deficiency may be manifested as fatigue, lethargy and mood changes and are shown through corkscrew hairs, perifollicular hemorrhages, gingival bleeding and anorexia [87]. If left untreated, it could result in pneumonia, which is one of the major complications and causes of death in scurvy [88].

Given the human body’s inability to synthesize vitamin C, dietary intake from fruits and vegetables is crucial for maintaining physiological functions. For optimal health in adults, a recommended reference intake is 75 mg/day for women and 90 mg/day for men [80]. Major dietary sources of vitamin C include guava, kiwi, citrus fruits, strawberries, chili peppers, kale and other brassica vegetables [89]. Inadequate intake of fruits and vegetables can adversely affect vitamin C status, as observed in individuals on restricted diets. Conversely, grains, legumes, nuts, seeds and animal products are poor sources of vitamin C, with minimal amounts found in meat (excluding liver), eggs and dairy [89].

The supplementation of vitamin C is associated with various impacts on human health. According to a meta-analysis by Aune et al., doses of vitamin C oscillating around 85–110 mg/day were related to a lower risk of coronary heart disease and stroke [90]. Moreover, twice the dose was associated with a lower risk of CVD mortality [91]. However, not all studies confirm these observations [92]. Some of them showed that doses of 125 mg/day and higher seem to have an influence on cardiovascular risk factors, such as lipid profile and glycemic control [93,94]. It also decreased HbA1c among patients with diabetes type 2 [94]. Vitamin C supplementation was also shown to reduce C-reactive protein concentration and the duration of the severe symptoms of the common cold [95,96].

### 5.3. Vitamin E

Vitamin E functions as the primary lipid-soluble antioxidant within the cellular defense system and is exclusively obtained from dietary sources. It appears as eight tocochromanols, four tocopherols (α-, β-, γ-, and δ-tocopherol) and four tocotrienols (α-, β-, γ-, and δ-tocotrienols), all of which possess antioxidant abilities [97]. The most crucial form is α-tocopherol, and it is the only one that meets the necessary human requirement for vitamin E [98]. However, a mixture of all tocopherols displayed a more effective inhibition of lipid peroxidation in comparison to α-tocopherol alone [99]. Recent studies are concentrated on the modulatory effects of vitamin E on various cellular processes, including the direct effect on T cell signal transduction, its membrane integrity, cellular pathways like NF-κB signaling and gene expression, concerning pro-inflammatory cytokines [31].

As an antioxidant, α-tocopherol plays a role in the protection of the integrity of polyunsaturated fatty acids (PUFAs) in cellular membranes and various plasma lipoproteins from OS [98], thereby preventing its peroxidation. By this action, PUFA can assist in upholding intracellular and cellular membrane stability and integrity. In addition, it takes part in vital functions such as erythrocyte stability and conductivity of nerves of both central and peripheral nervous systems [100].

The European Food Safety Authority Panel on Dietetic Products, Nutrition and Allergies suggested adequate intake of α-tocopherol: 13 mg/d for males and 11 mg/d for females [100]. However, the proposed amount may vary depending on the dietary content of PUFAs [101].

The main dietary sources of α-tocopherol include vegetable oils and their fat spreads, nuts and seeds, some fatty fish, egg yolk and whole grain cereals [100]. In particular, vegetable oils vary in their content of the different tocopherol forms: wheat germ, sunflower, olive and rapeseed oils are good sources of α-tocopherol, while wheat germ oil contains β-tocopherol. Soybean, corn, and rapeseed oils include γ-tocopherol and soybean oil encompasses δ-tocopherol [102].

When discussing vitamin E deficiency, there should be a distinction between congenital predisposition and dietary causes. The genetic syndrome revolving around this matter is ataxia with vitamin E deficiency. It is an autosomal recessive disease caused by mutations in the tocopherol transfer protein gene. The patients born with this disease absorb vitamin E normally, but due to a lack of tocopherol transfer protein, the serum vitamin E level is low [103]. The symptoms usually manifest between 5 and 15 years of age and present as dysarthria and progressive cerebellar ataxia [104]. Those patients may also develop eye disorders like retinitis pigmentosa and macular degeneration [105].

The symptoms of vitamin E deficiency caused by severe malnutrition include progressive peripheral neuropathy, difficulty in walking, tremors, a poor sense of balance, weakness of the muscles, retinal damage leading to vision problems, infertility and dementia [106]. Apart from these symptoms, deficiency can also lead to hemolytic anemia and neurological manifestations [107].

In cardiovascular contexts, vitamin E plays a significant role in preventing PLTs’ aggregation and the oxidation of low-density lipoproteins (LDL). It also decreases the uptake of LDL particles by macrophages [108]. Tocopherol has also been implicated in improving cardiovascular function by enhancing the activity of NOS, thereby promoting the production of vasodilatory NO [109].

## 6. Correlation between Vitamins and Blood Pressure Levels

Numerous studies have been conducted to elucidate the efficacy of vitamin supplementation in preventing HT. Table 1 compiles meta-analyses that confirm the antihypertensive effects of antioxidant vitamin supplementation.

### 6.1. Vitamin A and Carotenoids

Research on the association between vitamin A, carotenoids and HT has been scarce. Investigations have focused on the effects of vitamin A and carotenoid supplementation and plasma levels on the development of HT and changes in BP values. The data were inconclusive, and the results varied. Some studies indicated an inverse correlation between dietary vitamin A and BP levels [114], while others reported no significant association [115]. It is also important to mention that many studies were conducted on the Asian population.

Research conducted on the United States population by Li et al. pointed out that increased intake of carotenoids, including α-carotene, *β*-carotene, *β*-cryptoxanthin, lycopene, lutein and zeaxanthin, may lead to a lower prevalence of HT. The survey included 17,398 adults over 20 years old, excluding pregnant and lactating women and people with extreme dietary total energy intake. The beneficial effect of a lower risk of HT was particularly significant when total carotenoid dietary intake was above 100 μg/kg per day. For total carotenoids, *β*-cryptoxanthin, lycopene, lutein and zeaxanthin intake, the variables including age, sex, race, education, household income, smoking and drinking status, daily energy and fat intake, work and recreational activity did not have a significant impact on the association between the vitamin intake and HT. However, participants’ daily intake of vitamins was calculated based on their diet through the US Department of Agriculture Food and Nutrient Databases for Dietary Studies, using the “What’s in the Foods You Eat” search tool. Therefore, other factors connected to diet might have affected the results of the survey. Nevertheless, an inverse linear correlation between total daily carotenoid intake and prevalence of HT was observed [41].

The association between dietary vitamin A intake and the incidence of new-onset HT was deepened in a prospective cohort study conducted in China. The study utilized data from the China Health and Nutrition Survey and included 12,245 participants. The participants were men and non-pregnant women over the age of 18 without a history of HT. The median duration of follow-up was 6.1 years, during which dietary nutrient data intake was assessed using three consecutive 24h dietary recalls combined with a household food inventory. Throughout the study, 4304 (35.1%) of participants developed HT. Moreover, the analysis revealed that higher vitamin A intake led to a lower risk of HT development, which was described as an L-shaped relationship between vitamin A intake and the risk of HT. Comparing the lowest quartile of vitamin A intake (<227.3 μg RE/day) to the quartiles 2–4 (≥227.3 μg RE/day), a significantly reduced risk of new-onset HT was observed in a group with a higher vitamin A intake (adjusted HR, 0.73). Variables including sex, age, baseline SBP, smoking status and macronutrients as well as sodium and potassium intake did not significantly modify the association between dietary vitamin A intake and HT development. A stronger inverse association between vitamin A intake and new-onset HT was found in participants with BMI < 24 and dietary sodium to potassium intake ratio < 2.3. However, these results may not have significant clinical implications [116].

The influence of vitamin A supplementation on new-onset HT was also studied in double-blind, placebo-controlled, randomized, clinical trials including 955 HIV-positive pregnant women. Not only was the study examining the intake of multivitamins compared to placebo but also the intake of vitamin A alone. Supplementation comprising of thiamine (20 mg), riboflavin (20 mg), vitamin B_6_ (25 mg), vitamin B_12_ (50 μg), vitamin C (500 mg), vitamin E (30 mg), folic acid (0.8 mg) and a vitamin A supplement (30 mg β-carotene plus 5000 IU preformed vitamin A) managed to reduce the risk of hypertension by 38% (RR = 0.62, 95% CI; 0.40, 0.94; *p* = 0.03). Furthermore, researchers examined the outcomes of supplementation of β-carotene in doses of 30 mg plus 5000 IU preformed vitamin A. However, in this situation, the risk of HT was unchanged compared to the placebo group (RR = 1.00; *p* = 0.98). The limitation of this study was the fact that BP was not measured by the international standard of two readings 4 h apart and instead was measured only once [117].

Other studies showed no correlation between BP and vitamin A intake or even showed a positive correlation between supplementation of β-carotene and overall risk of CVD. A study by Llopis-González et al. was conducted on the adult population in the Spanish province of Valladolid. The study showed that vitamin A intake is higher in the population with HT, probably due to improvement in the dietary choices of hypertensive patients. However, after analysis, the researchers could not identify an association between fat-soluble vitamin intake, including vitamin A, and HT [118]. A meta-analysis by Yang et al. analyzed 16 reports on β-carotene treatment with 182,788 individuals enrolled. The study showed no beneficial effects on CVD and even a 9% increased risk of CVD, but only in men, and a 4% increased risk of the overall CVD incidence. Moreover, in the adult population supplemented with β-carotene a 17% risk increment for total stroke was noted compared to the placebo group. The limitation of this study was that 14 out of 16 studies had a follow-up duration shorter than 10 years, which is too short to accurately estimate the health outcomes, as CVD is a chronic condition. The study results also have limited applications to nutrient-deficient populations [119].

The evaluation of the relationship between HT and vitamin A was also assessed by examining its plasma levels. Czernichow et al. in their post-hoc analysis of the French SU.VI.MAX study found an inverse correlation between β-carotene plasma levels and HT, but only in men. However, this difference could be attributed to higher baseline levels of vitamin C and β-carotene in women (10.5 µg/mL and 0.67 µg/L, respectively) compared to men (8.9 µg/mL and 0.48 µg/L, respectively) in this study. Additionally, men had longer physical activity durations (>1 h per day), higher alcohol consumption and higher BMI values at baseline, which could have impacted the results [114].

The study by Hozawa et al. investigated the relationship between serum carotenoid concentrations and the incidence of hypertension in the Coronary Artery Risk Development in Young Adults (CARDIA) study. The study investigated 4412 participants from the United States for over 20 years period and at years 0, 7 and 15 measured the relationships between the sum of patients’ serum carotenoids level with incident HT. The research found a significant inverse association between the sum of four serum carotenoids, α-carotene, β-carotene, lutein/zeaxanthin, and cryptoxanthin, with incident hypertension and SBP. The mean value of the sum of four serum carotenoid concentrations was 44.5 µg/L and the group of participants with the lowest BP (<119/<79 mmHg) had higher serum carotenoid levels than patients with higher BP. The inverse association between carotenoid levels and hypertension risk persisted after adjusting for potential confounders like age, sex, race, BMI, smoking status and physical activity. These results suggest that higher circulating carotenoid levels, which reflect a diet rich in fruits and vegetables, are linked to a lower risk of developing hypertension [120].

The association between vitamin A and HT was also investigated in Jing et al. research. A total of 3102 women at 12 weeks of pregnancy were examined and their vitamin A serum levels were measured. A physiological serum level of vitamin A is 0.3–0.7 mg/L. In the study, the participants presented their levels in a range from 0.44 to 0.52 mg/L. Pregnant women were divided into two groups, the first group with hypertensive disorder (266 patients) and the control, which consisted of 2836 healthy pregnancies. The study group comprised 110 women with gestational hypertension, 65 with preeclampsia, 78 who had pregnancy with chronic HT, and 13 diagnosed with chronic HT with preeclampsia. Mean levels of vitamin A in serum (mg/L) were recorded as follows: 0.46 (*p* = 0.055), 0.47 (*p* = 0.01), 0.47 (*p* = 0.006) and 0.52 (*p* = 0.002), respectively. The mean vitamin A concentration for the control group was 0.44 mg/L. The difference in vitamin A levels between the control group and the hypertension types was statistically significant, except for the gestational HT group (*p* = 0.055). In the hypertension cases, the level of vitamin A was higher than in the control group. The study revealed that high serum levels of vitamin A in early pregnant women positively correlate with higher BP levels [115]. However, another study showed that in women with preeclampsia, especially with severe preeclampsia, the serum vitamin level is lower than in the healthy control group [121]; therefore, the sources are inconclusive and further research is needed.

### 6.2. Vitamin C

Another antioxidant vitamin exhibiting potential BP-lowering abilities is vitamin C.

An international meta-analysis conducted by Mason et al. observed that vitamin C supplementation, in doses ranging from 200 to 3000 mg/day, has a beneficial effect on BP levels in patients with type 2 diabetes. It decreased both SBP and DBP by 6.27 mmHg (*p* = 0.0002) and 3.77 mmHg (*p* = 0.002), respectively. Vitamin C concentration, age, BMI and duration of diabetes did not significantly influence the effectiveness of supplementation. As some of the patients were administering antihypertensive drugs, the efficacy of vitamin C as a stand-alone treatment for HT could not be elucidated and was rather suggested as an additional therapy [94].

A similar antihypertensive effect in type 2 diabetic patients was previously found in the analysis by Juraschek et al., where vitamin C supplementation decreased SBP by 4.71 mmHg (*p* = 0.63) and DBP by 4.07 mmHg (*p* = 0.08). What is more, in the general population, a 3.84 mmHg reduction in SBP (*p* < 0.001) and 1.48 mmHg reduction in DBP (*p* = 0.036) was observed. In subgroup analysis, the best results were observed in patients younger than 50 years old, in doses of vitamin C lower than 500 mg/day and with other antihypertensive interventions implemented [111].

A recent meta-analysis further supported the ability of vitamin C to reduce SBP by 3.0 mmHg (*p* = 0.001). The effect was more noticeable in patients previously diagnosed with HT (−3.2 mmHg, *p* = 0.002) or diabetic (−4.6 mmHg, *p* = 0.03) [122]. Contrarily, Guan et al. found no significant difference in SBP and DBP in hypertensive patients after vitamin C supplementation [110].

A study by Ward et al. analyzed the effects of vitamin C supplementation combined with polyphenols. The study revealed that treatment with 500 mg/day of vitamin C alone significantly reduced SBP by 1.8 mmHg compared to placebo (*p* = 0.03). Participants, who took only polyphenols had a reduction of SBP by 1.3 mmHg (*p* = 0.12); however, it was not statistically significant. On the contrary, patients who received both vitamin C and polyphenols had an increase of SBP by 4.8 mmHg versus placebo (*p* < 0.0001). These changes were unaltered after adjustment for age and sex. A direct correlation between vitamin C supplementation and BP levels could not be established [123].

The immediate effect of intravenous injection of vitamin C (IVC) on BP was also examined in an Australian study. Furthermore, 75 min after injection, patients who had received higher doses of IVC (>30 g) experienced a more pronounced reduction in both SBP and DBP values (−6.2 and −7.1 mmHg, respectively) compared to those with lower doses (−1.7 and −1.6 mmHg, respectively). BMI significantly influenced SBP response to IVC treatment. In the overweight group, SBP decreased by 6.7 mmHg from a baseline of 129 mmHg. In contrast, the decrease in healthy-weight individuals was much smaller, with SBP dropping by only 1.6 mmHg from 114.9 mmHg. However, the response of DBP to IVC treatment showed an inverse relationship with weight. In the healthy-weight group, DBP decreased by almost 8 mmHg from 77.4 mmHg, while there was no significant change in the overweight/obese group. A worth-noting fact is that in prehypertensive participants SBP declined by a mean of 8 mmHg and DBP by 6 mmHg over the full duration of IVC treatment. In the normotensive group, the average difference was lower: −6.7 mmHg in SBP and −5.5 mmHg in DBP [124].

In addition to supplementation, plasma vitamin C levels were also found to correlate with BP. Particularly, participants with vitamin C serum levels of 68.1–191μmol/L had lower SBP and DBP by 1.84 and 2.08 mmHg, respectively, compared to those with concentrations ranging from 1.87 to 32.1 μmol/L. An inverse relationship between vitamin C and both SBP and DBP values was found and persisted after adjustments for sex, age, race, education level, BMI, pulse, alcohol consumption and smoking status. Moreover, a U-shaped relationship between vitamin C dose and SBP was found when adjusted to only females, and an inverted U-shaped association between vitamin C dose and DBP was found in males. The study suggested that this difference could have been a result of interactions between different sex hormones. A U-shaped pattern between vitamin C and both SBP and DBP was also observed in people over 50 years old, showing the most beneficial effect when vitamin C levels were over 40 μmol/L [125].

Comparable conclusions were observed in the meta-analysis of Ran et al., where hypertensive subjects had their concentration of vitamin C lower by approximately 15.13 μmol/L than normotensive ones. The study found that serum vitamin C concentration exhibits a noteworthy inverse correlation with both SBP (*p* < 0.00001) and DBP (*p* < 0.00001) and it remained the same after adjustment for sex, age and BMI. Subgroup analysis revealed that hypertensive subjects consistently taking antihypertensive drugs had significantly lower serum vitamin C levels (15.97 μmol/L). This finding suggested that antihypertensive drugs may deplete serum vitamin C [126].

### 6.3. Vitamin E

Recent research has shed light on the potential role of vitamin E in the improvement of BP levels. However, determining its precise impact on BP and the optimal dosage remains a subject of investigation across various studies.

The research conducted by Zhang et al. was the first one to dive into the prospective relationship of dietary vitamin E intake with new-onset HT [127]. The study enrolled 12,177 patients with a mean age of 41 years. Their medium BMI was approximately 22 kg/m^2^. Nearly 30% of them were smokers and above 30% were consuming alcohol in their daily lives. Individuals with higher vitamin E consumption were generally older, female and did not smoke or drink alcohol. In addition, their BMI, SBP and DBP were higher as well as their intake of fat, potassium, sodium, fruits and legumes. However, they consumed less vitamin A, vitamin C, vegetables and grains. After a 6.1-year follow-up, the study revealed that 4269 (35.1%) individuals developed HT. The reverse J-shaped association (*p* for nonlinearity < 0.001) between dietary vitamin E intake and the onset of HT was noted, with the minimal risk of HT ranging from 18.75 to 40.53 mg/day for vitamin E intake [127]. The mentioned variables were included in the analysis and did not change the relationship between dietary vitamin intake and the development of essential HT.

The re-analysis of the National Health and Nutrition Survey, which gathered 1405 males and 2102 females, also supported the protective effect of a dose ranging from 10–40 mg/day [128]. Doses above 10 mg/day of supplemented vitamin E were associated with a lower prevalence of HT, with odds ratios of 0.81. Although the intake of vitamin E was linked to a reduced risk of HT (*p* = 0.01), it is important to note that individuals with higher vitamin E intake (average 11 mg/day, *p* < 0.001) also had higher than average intakes of vitamin C, potassium and magnesium, all of which have potential BP-lowering effects [128]. Moreover, those patients were associated with favorable lifestyle factors such as a lower percentage of alcohol consumption (77% of them were consuming less than 20 g per day) and a lower percentage of smokers (32%). After adjustment for those covariates and others (age, sex, BMI, physical activity, intakes of energy, n-3 fatty acid, saturated fatty acid, dietary salt, dietary fiber and vitamin C), the antihypertensive effect was sustained, but it should be noted that some additional confounding variables, related to a generally healthy lifestyle, might have influenced the results.

Similarly, in their meta-analysis, Emami et al. showed that supplementation with lower doses of vitamin E had a better effect on SBP than higher doses (above 400 mg/day) [50]. This decrease in the antihypertensive effect with increasing vitamin E doses was also noted in the study by Zhang et al. [127]. Both studies concluded that vitamin E showed better effects on SBP reduction in the short term (up to 8 weeks) with a minimum dose of 80 mg/day, while in the duration of 6 months, the most significant BP reduction was noted with doses ranging from 18.75 to 40.53 mg/day. Moreover, vitamin E significantly decreased SBP by 3.4 mmHg (*p* < 0.001), while no effect on DBP and mean arterial pressure was observed. The largest decreases were recorded in people with chronic diseases, such as type 2 diabetes and HT, in which oxidative stress is increased [50]. In comparison to healthy individuals, NO bioavailability of patients with chronic diseases is more likely to be limited. The increase of NO bioavailability caused by the supply of antioxidants may explain the results in an improvement in BP parameters [129]. It can also clarify the non-significant effects of vitamin E on DBP and MAP, on which NO does not influence.

Another study was conducted to examine the long-term effects of vitamin E intake, measured over several years [130]. The supplementation involved doses lower than 10 mg per day. The study by Mishra et al. was conducted to determine whether dietary vitamin E intake during childhood or mid-life could predict adult HT. Initially, in 1950, dietary information was collected from 2980 individuals aged 4 through a questionnaire. The same participants were surveyed again in 1989, at the age of 43, to reassess their dietary intake, and their BP was measured. The findings revealed that individuals with the lowest vitamin E intake during both childhood (2.2 mg/day) and adulthood (5.3 mg/day) exhibited a higher likelihood of HT (OR 1.78) compared to those with high consumption levels at both stages of life (3.2 mg/day in childhood and 13.1 mg/day in adulthood). The odds ratio for hypertension was adjusted for BMI at age 43 years as well as dietary intake of calcium, sodium, potassium and magnesium in both childhood and adulthood. However, in the group with the lowest vitamin E intake during childhood and adulthood, the majority (58.4%) of participants were male and smokers (39.5%); only 43% exercised more than once a week, but in the highest third intake in both life stages, females constituted most (57.1%) of the group and only 22.8% was smoking. In the final stage of the study, around 353 of the 2980 individuals (12%) were diagnosed with HT at the age of 43, with men being 1.71 times more likely to develop HT than women [130]. Still, vitamin E intake seemed to have a strong impact on HT onset, independently of sex, smoking, physical activity and occupational social class.

A lower risk of HT was seen in the majority of the ethnic groups, with Chinese participants being the most studied. Lower BP was also observed in the British population as well as Iranian ones [130,131]. However, some studies showed that there may be differences in BP reduction by population, as Spain individuals did not show any association between HT and vitamin E intake [132]. Further studies in various ethnic groups are needed as they can influence the results.

A recent study carried out by Kumar et al. dived into the influence of vitamin E on cardiovascular diseases. The research indicated that using vitamin E supplements for more than four years led to an impressive 59% reduction in deaths from coronary heart disease [36].

Behers et al. analyzed vitamins C, D and E and minerals for BP reduction in the normotensive population. Out of all vitamins, only vitamin E provided a statistically significant reduction in SBP of −1.76 mm Hg [133].

Multiple studies provide evidence supporting the idea that vitamin E may have a positive impact on preventing HT and reducing BP, particularly SBP. However, the optimal dosage for achieving these effects is still under evaluation.

### 6.4. Combinations of Vitamins

Several studies have focused on evaluating the combined effects of supplementing various antioxidant vitamins.

Czernichow et al. conducted an analysis using data from the SU.VI.MAX randomized primary prevention trial. They enrolled a cohort of 5086 patients to evaluate the effects of nutritional doses of antioxidant vitamins and trace elements on the 6.5-year risk of HT. The subjects from the control group received daily pills containing low-dose antioxidants: 120 mg of vitamin C, 30 mg of vitamin E and 6 mg of β-carotene, along with 100 mg of selenium and 20 mg of zinc. Multivariate analysis was adjusted for factors that may influence the development of hypertension: age, physical activity, BMI, education level, smoking habits and alcohol consumption. No statistically significant outcomes were observed regarding the effects of supplementation on the risk of hypertension in both men and women. However, it is important to note that the initial BP measurements in this study were taken one year after the baseline, potentially affecting the results [114]. This timing issue may explain the absence of observed reductions in HT risk, as the Dietary Approaches to Stop Hypertension trial, which used similar antioxidants and doses, demonstrated notable BP reductions and increased antioxidant levels after just several weeks of intervention [134].

A different study indicated that the supplementation of certain antioxidants in a population with a micronutrient-poor diet might help prevent HT. In this study, participants with a mean age of 54 and cytological diagnosis of esophageal dysplasia took daily doses of vitamin A, vitamin C, vitamin E and 23 other supplements for six years, at levels two to three times higher than the US Recommended Dietary Allowance. It is important to note that approximately 66% of the men were smokers, while only a small number of women smoked. Additionally, 33% of men and 8% of women reported drinking alcohol in the past year, typically in small amounts and on rare occasions. Therefore, the analyses were conducted separately for each sex and included factors such as SBP and DBP, age, BMI, drinking and smoking habits. The results showed a reduced prevalence of HT, with a more pronounced effect in men (RR for men = 0.43, RR for women = 0.92). For men, the treated group had an average end-of-trial DBP 2.02 mmHg lower (*p* = 0.009) and SBP 1.33 mmHg lower (*p* = 0.32) than the control group. For women, the differences were smaller, with the treated group having a DBP 0.41 mmHg lower (*p* = 0.58) and SBP 0.74 mmHg lower (*p* = 0.53), but the results were not statistically significant [135].

A study conducted by Wu et al. investigated the effects of six antioxidants—vitamins A, C and E, as well as manganese, selenium and zinc—on HT. The research explored the correlation between HT and a composite dietary antioxidant index (CDAI), which is a comprehensive score of multiple dietary antioxidants. In the study, a linear negative correlation between CDAI and hypertension has been stated in the adult population. The findings revealed that individuals in the highest quartile of CDAI (OR = 0.81; *p* = 0.006) exhibited a lower likelihood of elevated BP (mean BP: 116.9/67.6 mmHg). Conversely, those in the lowest quartile of CDAI showed an adverse effect, with an increased risk of HT (mean BP = 152.5/76.1). The authors developed three models to analyze the data with adjustments for various covariates. Model 1, which included no covariates, yielded an OR = 0.97 and *p* < 0.001. Model 2 adjusted for sex and age, resulting in an OR of 0.99 with a *p* = 0.019. Model 3 was further adjusted for sex, age, alcohol consumption, smoking, physical activity, race, education, daily energy intake and BMI, showing an OR = 0.98 and *p* = 0.016. A 3% reduction in HT risk per unit increase in CDAI remained consistent across all models [136].

A recent meta-analysis by Qi et al. investigated the effects of vitamins B_2_, C, D, E and folic acid on patients with HT involving 2218 patients with a mean age of 59. Females constituted the majority of the group (64%). The results showed that only vitamin E significantly lowered SBP (mean difference: −14.14 mmHg) compared to placebo. No effects were found for any of the five vitamins on DBP, 24h SBP and DBP or heart rate. However, the study found that combining vitamin C with vitamin E resulted in a reduction in 24h DBP. Moreover, the authors emphasized that the antihypertensive effects of vitamins are dose-dependent. They also suggested that the results may vary by sex and geographic region, as seven out of eight European studies found no significant impact of vitamin intervention on BP, while all West Asian ones reported a significant effect. The study, however, had major limitations due to high heterogeneity, low evidence credibility and the inclusion of only a few studies. Additionally, confounders such as smoking and alcohol intake were also not included in the analysis. The authors advised caution in interpreting the results and highlighted the need for future research [113].

Moreover, vitamin supplementation is found to positively impact cardiovascular diseases, which are one of the consequences of untreated HT. Research from 2023 conducted by Kumar et al. has noticed that a significant decrease in the incidence of ischemic heart disease, between 10% and 14%, was seen in women who took vitamin C, E and A supplements [36].

To summarize, the efficacy of individual vitamin supplementation in lowering BP remains uncertain. The most promising approach appears to involve supplementing with vitamin C, vitamin E or a combination of antioxidant vitamins, particularly in populations with micronutrient deficiencies. Higher overall consumption of these vitamins seems to have a positive effect on reducing BP. While studies conducted on animals have demonstrated the beneficial impact of vitamins on BP regulation, clinical trial results have been inconsistent. Therefore, further research is essential to clarify these findings and establish the optimal use of vitamin supplementation for HT prevention and treatment.

### 6.5. The Influence of Gender on Blood Pressure

The influence of sex on blood pressure in the context of antioxidants, particularly vitamins A, C and E, has been explored in different studies with various results. Research on the relationship between vitamin A and hypertension has been inconclusive, with findings showing both inverse and nonsignificant correlations. In one study, a statistically decreasing trend in the hypertension risk was found only in women, when vitamin A was supplemented together with vitamin C [114]. However, in a study by Llopis-González et al., despite the minor difference in vitamin A intake between men and women (≥58.3 μg/dL and <49.5 μg/dL, respectively), the sex did not influence the final results [137]. Moreover, in Li et al. and Zang et al., sex and other variables did not have a significant impact on the association between vitamin intake and HT [41,115].Studies from the U.S. and China suggested that a higher intake of carotenoids and vitamin A might lower the risk of hypertension, regardless of sex [116]. Vitamin C has demonstrated blood pressure-lowering effects in diabetic patients and the general population, with some studies indicating a more significant impact in men and at specific dosages, 60 μmol/L [111]. However, other studies found that the results were unchanged after adjustment for sex [123]. Several studies have shown an inverse relationship between plasma vitamin C levels and BP, but this relationship varied by sex and age. Specifically, a U-shaped association between vitamin C dosage and SBP was found in females, while an inverted U-shaped relationship between vitamin C dosage and DBP was observed in males. These findings highlight sex-specific differences in the optimal dosage of vitamin C for blood pressure regulation [125]. However, regarding the risk of hypertension and serum concentration of antioxidative molecules in normotensive men, no association was observed with vitamins C and E or zinc. The reduction in hypertension risk with higher vitamin C levels in women was of borderline statistical significance [114]. Vitamin E intake exhibited a reverse J-shaped association with hypertension, where optimal dosages reduced the risk. Studies found that higher vitamin E intake (more than 80 μg/day) is often linked to a healthier lifestyle, no smoking, no alcohol consumption, and a low sodium diet. Long-term studies indicated that vitamin E intake during childhood and adulthood could reduce hypertension and stroke mortality in the future. These benefits were more significant in men compared to women [130]. Mark et al. found that for men, the average diastolic blood pressure was 2.02 mmHg lower in the treated group than in the control group (*p* = 0.009), and the average systolic pressure was 1.33 mmHg lower (*p* = 0.32). For women, the differences in blood pressure between the two groups at the end-of-trial examinations were smaller, 0,41 mmHg (*p* = 0.58) for DBP and 0.74 mmHg (*p* = 0.54) for average systolic blood pressure [135].Kumar et al. noticed that females have greater knowledge and concern over their health and body and that may be the reason for women to supplement vitamins more frequently and regularly than men [36]. Overall, the influence of sex, as well as other factors on the relationship between antioxidants and blood pressure remains complex and requires further investigation. It is worth mentioning that HT is a complex multivariable pathology, which makes the influence of sex on this disease difficult to state.

## 7. Conclusions

Oxidative stress is an important factor that plays a role in HT development. ROS, such as O_2_-, H_2_O_2_ and ONOO- contribute to BP elevation by promoting endothelial damage and vasoconstriction, leading to increased arterial resistance. Additional mechanisms, including decreased glomerular filtration rate, reduced natriuresis and the production of certain cytokines by immune cells, also contribute to elevated BP.

Organisms have developed an antioxidant defense system that neutralizes ROS and mitigates the damage they cause. This system includes nonenzymatic antioxidants like vitamins A, C and E. They combat OS by altering certain gene expression levels and, through an increase in NO production, a molecule with vasodilatory properties. These vitamins can be obtained from various nutritional sources, with the highest concentrations found in fruits and vegetables. Vitamin A is abundant in carrots and spinach; vitamin C is present in citrus fruits and broccoli; and vegetable oils are rich in vitamin E. Deficiencies of these vitamins can lead to severe complications. However, they can also be supplemented to ensure adequate intake.

Various factors contribute to the development of HT, which can be categorized into modifiable and non-modifiable risk factors. Modifiable risk factors include BMI, alcohol consumption, smoking status, sodium and nutrient intake and physical activity. Vitamin intake and serum levels seem to have an impact on BP values as well. As all of the variables mentioned could affect the association between BP values and vitamins, the results were adjusted for these additional confounders.

The latest *Guidelines for the Management of Patients with Chronic Coronary Disease* published in 2023 for the United States point out that additional supplementation of vitamins should not be used in order to decrease mortality in coronary heart disease. The influence of non-prescribed vitamin supplementation was discussed, particularly in the context of vitamins D and C, beta-carotene and multivitamins. These substances were claimed not to significantly reduce the frequency of cardiovascular events. Therefore, it was advised not to supplement them as a solution for managing coronary disease. However, the guidelines did not concern their role in the prevention of hypertension [138].

Moreover, ESH/ESC guidelines highlighted the influence of oxidative stress in HT development. One of the BP modifiers is proper diet, especially the Mediterranean one [139], which is well-known for its antioxidative properties, due to containing various antioxidants, including vitamin C, vitamin E and carotenoids [140]. This diet is proven to reduce all-cause mortality rates and lower incidences of cardiovascular diseases [139]. Numerous studies have investigated the impact of vitamin and carotenoid supplementation, dietary intake and serum levels on BP values and the risk of HT development. The French SU.VI.MAX study found an inverse correlation between β-carotene plasma level and HT, but only in men [116,137]. Vitamin C intake with a mean value of 757.5 mg/d for 6 weeks resulted in a decrease of 4.09 mmHg in SBP and 2.3 mmHg in DBP [122]. Additionally, individuals with vitamin C serum levels ranging from 68.1 to 191 µmol/L experienced reductions in their SBP and DBP by 1.84 and 2.08 mmHg, respectively [125].

For vitamin E, supplementation over more than four weeks led to a 14.14 mmHg reduction in SBP [113]. It is noteworthy that every 1mmHg decrease in SBP could potentially save up to 10,000 lives annually. Therefore, the supplementation of vitamins A, C and E appears to have a beneficial effect on BP regulation and HT prevention. On the other hand, a study conducted by An et al. stated that vitamins C and E do not affect cardiovascular diseases [141]. The results, however, could have been influenced by the short duration, small sample sizes and significant heterogeneity of some studies, as well as the potential inaccuracies in assessing vitamin intake. These limitations highlight the need for more robust and comprehensive research to conclusively determine the impact of vitamin supplementation on BP and HT management. Future studies should aim for larger cohorts, longer follow-up periods, and precise dietary assessments to provide clearer insights. Establishing a definitive link between vitamin intake and BP regulation could lead to more effective nutritional interventions for preventing and managing HT in the future.

## Figures and Tables

**Table 1:** Meta-analyses’ comparison of vitamin supplementation impact on blood pressure reduction in the general population.

Name of Study	Vitamin Type	No. of Patients	No. of Trails	Range of Vitamin Doses	Duration of the Study	Study Results
Guan et al. (2020) [110]	Vitamin C	614	8	300–1000 mg/day	4 to 24 weeks	Reducing SBP by 4.09 mmHg (95% CI: −5.56, −2.62; *p* < 0.001) Reducing DBP by 2.30 mmHg (95% CI; −4.27, −0.331; *p* = 0.02)
Juraschek et al. (2012) [111]	Vitamin C	1407	29	60–4000 mg/day	2 to 26 weeks	Reducing SBP by 3.84 mmHg (95% CI: −5.29, −2.38 mmHg; *p* < 0.01) Reducing DBP by 1.48 mmHg (95% CI: −2.86, −0.10 mmHg; *p* = 0.04)
McRae et al. (2006) [112]	Vitamin C	284	13	400–2000 mg/day	4 to 12 weeks	Reducing SBP by 3.9 mmHg (95% CI; −13.0, 6.0) *
Emami et al. (2019) [50]	Vitamin E	839	18	80–1206 mg/day	3 to 48 weeks	Reducing SBP by 3.4 mmHg (95% CI; −6.7,−0.1; *p* < 0.001)
Qi et al. (2024) [113]	Vitamin E	2218	23	135–600 mg/day	More than 4 weeks	Reducing SBP by 14.14 mm Hg (95% CI; −27.62,–0.88)

* Only 7 out of the 13 researches achieved statistically significant reductions in systolic blood pressure. SBP—systolic blood pressure; DBP—diastolic blood pressure; CI—confidence interval.

## Data Availability

The data used in this article are sourced from materials mentioned in the References section.

## Abbreviations

ACE	angiotensin-converting enzyme
ADMA	asymmetric dimethylarginine
Ang II	angiotensin II
BH4	tetrahydrobiopterin
BMI	body mass index
BP	blood pressure
CD	cluster of differentiation
CDAI	composite dietary antioxidant index
CI	confidence interval
CVD	cardiovascular diseases
DBP	diastolic blood pressure
DCs	dendritic cells
DDAH	dimethylargininedimethylaminohydrolase
DNA	Deoxyribonucleic acid
ESH/ESC	European Society of Hypertension/European Society of Cardiology
eNOS	endothelial NO synthase
GSH	glutathione
H_2_O_2_	hydrogen peroxide
HbA1c	glycated hemoglobin
HIV	Human immunodeficiency viruses
HR	hazard ratio
IL	interleukin
IsoLGs	isolevuglandins
IVC	intravenous injection of vitamin C
LDL	low-density lipoproteins
NF-κB	Nuclear factor kappa-light-chain-enhancer of activated B cells
NO	nitric oxide
Nox	nicotinamide adenine dinucleotide phosphate oxidase
OS	oxidative stress
O_2_	molecular oxygen
O_2-_	superoxide anion
ONOO	peroxynitrite
OR	odds ratio
PLTs	platelets
PUFAs	polyunsaturated fatty acids
RDA	recommended daily allowance
RE	retinol equivalents
REA	retinol activity equivalent
RNA	ribonucleic acid
ROS	reactive oxygen species
RR	relative risk
SBP	systolic blood pressure
TNF-α	tumor necrosis factor α
WMD	weighted mean difference
VAD	vitamin A deficiency
